# Chronology of Thyroid Cancer

**DOI:** 10.1007/s00268-022-06741-4

**Published:** 2022-09-24

**Authors:** Akira Miyauchi

**Affiliations:** Department of Surgery, Kuma Hospital, 8-2-35 Shimoyamate-dori, Chuo-ku, Kobe, Hyogo 650-0011 Japan

## Abstract

**Introduction:**

The basic nature of cancer includes unlimited growth, invasion, and metastasis. The TNM staging system is very simple and popular. It indicates the degree of the anatomical spread of the disease but does not include tumor growth. Collins reported that human tumors grow exponentially, which can be expressed in doubling time.

**Patients and Methods:**

We found that in patients with medullary thyroid carcinoma (MTC) and papillary thyroid carcinoma (PTC) serum calcitonin and thyroglobulin levels changed exponentially over time, respectively, and that doubling times of these values were very strong prognostic factors. Doubling time has two major limitations. Doubling rate resolves these limitations. Using doubling rate, we performed kinetic analyses on tumor volume during active surveillance of micro-PTC.

**Results:**

Our kinetic studies on patients with biochemically persistent disease revealed that 17% of MTC and 51% of PTC showed decrease in serum tumor marker levels over time. During active surveillance of micro-PTC, 17% of the patients showed clear decrease in their tumor volume. The evidences currently available are limited. However, our data indicate the following: Growth slowdown and regression are very common phenomena in the natural history of micro-PTC, clinical PTC in young and middle-aged patients, and hereditary MTC. The biologic characteristics of cancers of the same name, such as PTC, are diverse and vary widely with age.

**Conclusions:**

Doubling time and doubling rate are very powerful tools to provide the most appropriate management for the patients with thyroid cancers. Knowing the natural history of thyroid cancer is essential for the best disease management of thyroid cancer.

## Introduction

Prof. Sugitani, thank you for the kind introduction. Members and guests. I have had the great honor to serve President of International Association of Endocrine Surgeons for these three years, and today, I have the privileged opportunity of Presidential Address. My talk for you is “Chronology of Thyroid Cancer.” Chrono- means time, and -ology means learning. Here, I use chronology as an area of learning on time-related phenomena.

The basic nature of cancer includes unlimited growth, invasion to surrounding tissues, and metastasis to lymph nodes and distant organs. When I first learned the TNM staging system, I thought that it contained all these features and was very simple and beautiful. In the older versions, in patients with papillary or follicular carcinoma 45 years and older, the stage was determined with T, N and M. Quite clear. However, all patients younger than 45 years without distant metastases were classified as Stage I, and patients with distant metastases were classified as Stage II instead of Stage IV. I thought this was strange. In the current 8th edition, the age limit is set to 55, but the system is basically unchanged [1].

I learned that this was due to the fact that younger patients had a high incidence of recurrence but a low cancer mortality, and that cancer mortality increased after middle age as nicely demonstrated by Mazzaferri and Jhiang [2]. Later, Mazzaferri and Kloos [3] added the incidence of distant metastasis, which showed U-shape change over age of the patients. Increase in locoregional recurrence, distant metastasis and cancer mortality with older ages was easy to understand. However, high incidence of distant metastasis and low mortality in younger patients was confusing. Was it because surgeons performed good surgery? Radioactive iodine (RAI) was very effective? TSH suppressive therapy was effective? These might have been true, but can these explain all? There might have been other reasons.

## Kinetic analysis of change in tumor volume over time

**Fig. 1:**
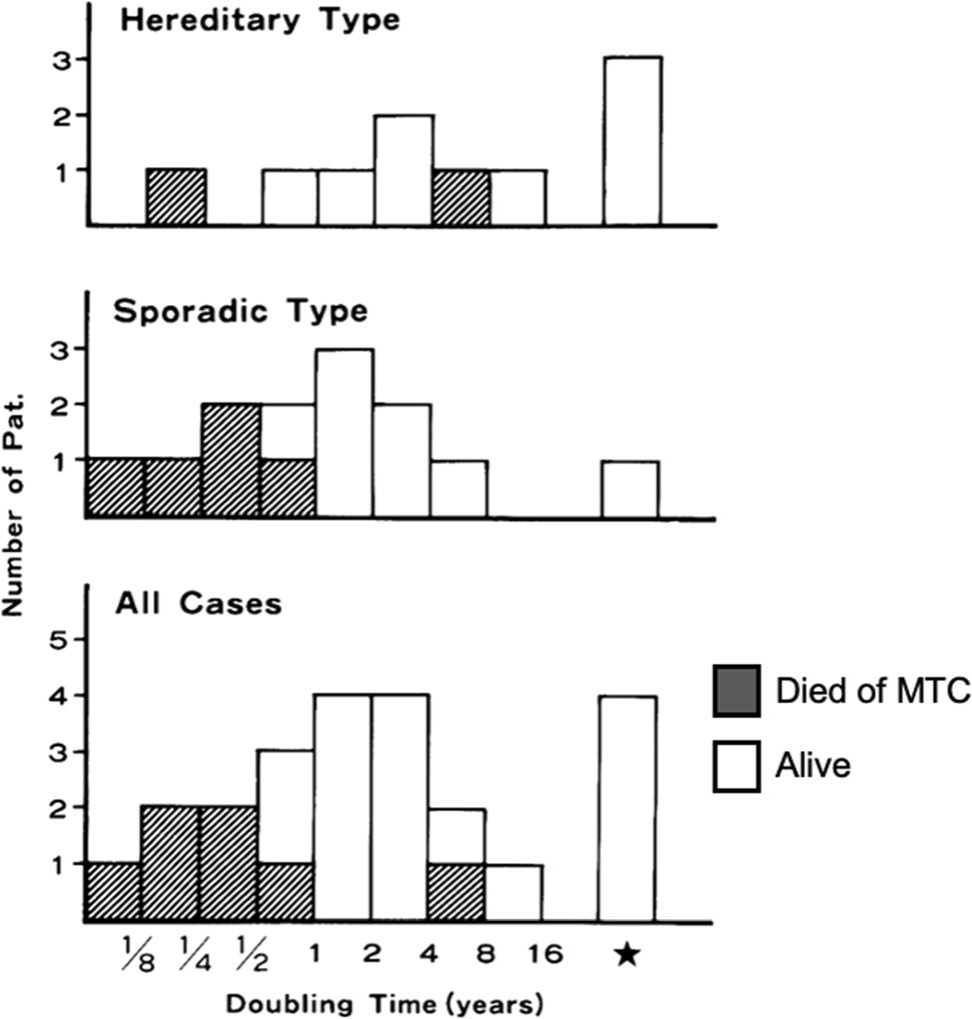
Distribution of patients with medullary thyroid carcinoma according to calcitonin doubling time and survival. Solid column represents deceased patients. : patients with negative doubling time values. (Cited from Miyauchi et al. [5], with permission)

The TNM staging system is very simple and easy to use. However, it merely indicates the degree of the anatomical spread of the disease. In my opinion, its major limitation is that it does not include tumor growth or disease progression. Collins reported that growth of human tumor is exponential [4]. On a semi-log graph taking log of tumor volume or tumor cell number on the vertical axis and time on the horizontal axis, the growth is displayed in a straight line. In 1984, we discovered that changes in serum calcitonin levels in patients with medullary thyroid carcinoma (MTC) with postoperative persistent hypercalcitoninemia, biochemically persistent disease (BPD), were exponential [5]. This was consistent with the Collins’ tumor growth model. Patients with steep slopes died of the disease. We calculated calcitonin doubling time (DT) with the slope of the regression line. The distribution of calcitonin DT was very wide and patients with short DT died of the disease (Fig. 1). We reported that calcitonin DT was a strong prognostic factor of MTC. Twenty-one years after our report, Barbet et al. reported that calcitonin and carcinoembryonic antigen DTs were very strong prognostic factors of MTC [6]. They showed that on multivariate analysis, only the inverse of calcitonin DT was the independent prognostic factor. Giraudet et al. [7] presented a similar report, and the American Thyroid Association guidelines on the management of MTC adopted calcitonin DT as a strong prognostic factor [8].

**Fig. 2:**
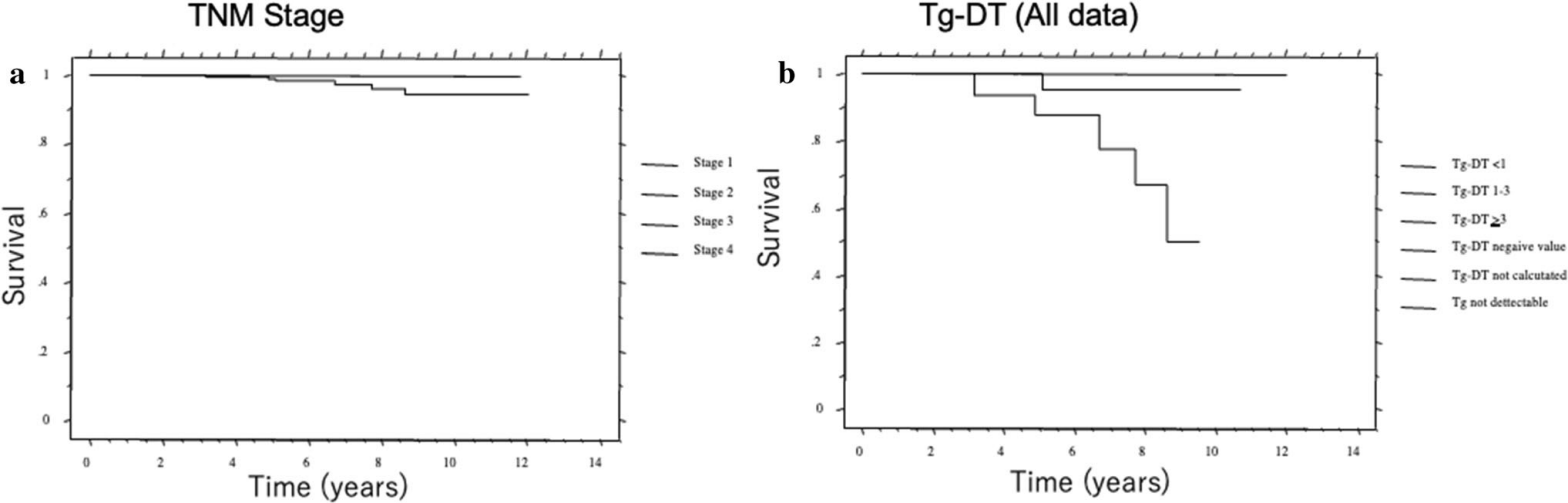
Kaplan–Meier cause-specific survival curves according to TNM stage (**a**) and thyroglobulin doubling time (Tg-DT) (**b**) in patients with papillary thyroid carcinoma. (Cited from Miyauchi et al. [9], with permission)

Papillary and follicular thyroid carcinoma are derived from thyroid follicular cells and produce thyroglobulin. Therefore, serum thyroglobulin could be an excellent tumor marker of these carcinomas. However, normal thyroid tissue produces thyroglobulin, presence of thyroglobulin antibody interferes thyroglobulin measurement, and serum thyroglobulin values depend on TSH levels. To overcome these limitations, we selected patients with papillary thyroid carcinoma (PTC) who underwent total thyroidectomy, having negative thyroglobulin antibody test results, and serum thyroglobulin values measured under TSH suppression. On semi-log graphs, serum thyroglobulin levels in individual patients changed linearly over time [9]. We calculated thyroglobulin DT for each patient. Thyroglobulin DT discriminated the prognosis more clearly than the TNM stage (Fig. 2) [9]. On univariate analysis, many classical prognostic factors were significant. However, on multivariate analysis, only thyroglobulin DT remained as an independent predictor of disease-specific survival, distant metastases and locoregional recurrence [9].

Tumor marker DT is a very strong prognostic factor. Using this, quantitative estimation of disease progression and prognosis in individual patients is possible. If the cancer remains after surgery, the remaining tumor grows at the same growth rate as before surgery and leads to death. Generally, 1000 g tumor might kill the host [4]. Assuming an ideal tumor marker in which the blood tumor marker value is directly proportional to the tumor volume, the remaining tumor volume can be calculated with the resected tumor volume and the preoperative and postoperative blood tumor markers [10]. If the number of doublings required for the residual tumor to reach 1000 g is *β*, the period until death is expressed as βDT, a very simple formula [10]. This is possible if there is a very sensitive marker such as calcitonin in MTC.

**Fig. 3:**
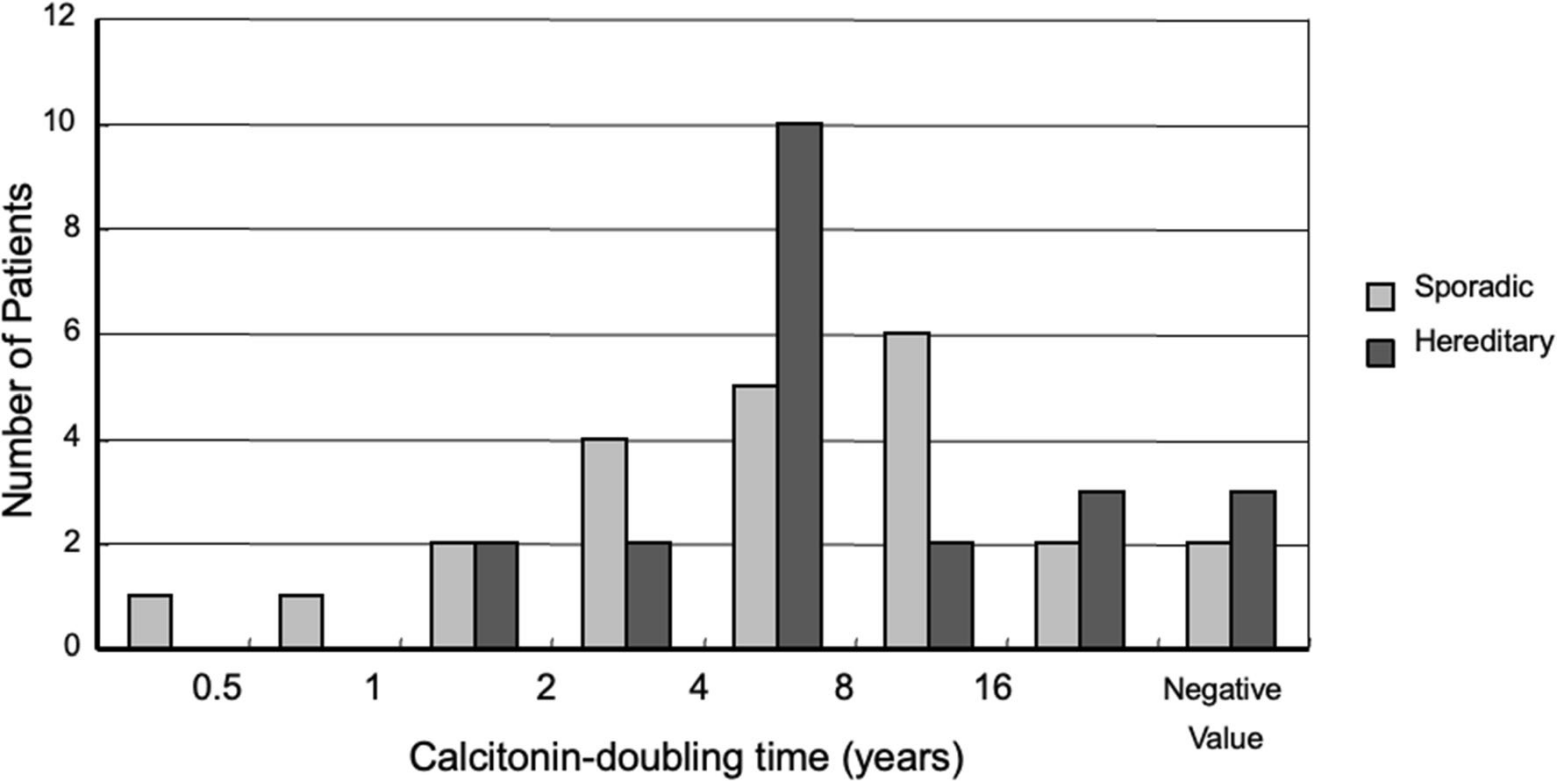
Distribution according to calcitonin doubling time of 45 patients with medullary thyroid carcinoma who underwent surgery at Kuma Hospital and had persistent hypercalcitoninemia postoperatively

This is an example. A 66-year-old man with 1.8 cm sporadic *RET* negative MTC underwent surgery. Pathology showed many microscopic lymph node metastases. Preoperative calcitonin at 3800 pg/ml decreased to 190 pg/ml postoperatively, indicating he had BPD. How do you predict the prognosis of the patient? Pathological node metastases and persistent disease. Poor prognosis? With the resected tumor weight, preoperative and postoperative calcitonin values, the residual tumor weight was calculated to be 0.18 g. The number (*β*) of doublings for this tumor to reach 1000 g was 12.4. Calcitonin DT was widely distributed in 45 patients with MTC treated at Kuma Hospital who had BPD postoperatively (Fig. 3). Most of the patients had calcitonin DT of longer than 4 years. If his calcitonin DT was 4 years, the expected survival was calculated to be 49 years. If 1000 g seems too big, you can set the host-killing tumor size to 100 g. In this setting, *β* is 9.1 and the expected survival is 36 years. The actual calcitonin DT calculated 3 years after surgery was 5.6 years. I can say that this patient will not die of MTC although he has persistent disease. I said to the patient, “Don’t worry too much, you will live for long time.”

Another example is a 78-year-old woman with a 1.3 cm pulmonary metastasis of PTC. RAI was ineffective. Her serum thyroglobulin level increased gradually over time. Thyroglobulin doubling time was 6.4 years. Using the doubling time and the current tumor size, the tumor size after 10 years was estimated to be 1.9 cm. This is unlikely to cause symptoms. She will be doing well at least for 10 years although she has RAI refractory pulmonary metastasis. Thus, our tumor board did not recommend tyrosine kinase inhibitor to her at this time.

As I explained, doubling time is a very useful tool to provide the best management for the patients with thyroid cancer in cancer clinics. For your convenience, we created a calculator. You can download it for free from Kuma Hospital website at https://www.kuma-h.or.jp/english/.

## Spontaneous deceleration in growth rate and regression

From now on, I would like to look at different aspects of tumor volume kinetics. In our first reports on calcitonin DT and thyroglobulin DT, 17% of patients with MTC (Fig. 1) and 16% of patients with PTC (Table 1), respectively, had tumor marker DT of negative values [5, 9]. Among 137 patients of PTC with BPD, as many as 69 patients (50.4%) had negative thyroglobulin DT values. I was confused how to handle these negative values. In Fig. 1, the patients with negative DT values should have appeared left of point 0 mathematically, but that didn’t make sense, because these patients had excellent outcomes. Negative values of DT cause a problem of discontinuity between positive values. In addition, I had the following questions: Was it due to assay variations, due to decrease in production of calcitonin and thyroglobulin, or due to decrease in tumor volume? Regarding the discontinuity problem, Barbet offered a smart solution. He used the inverse of DT for statistical calculations [6].

**Table 1:** Distribution of the patients with papillary thyroid carcinoma according to thyroglobulin doubling time status calculated using all available data and using only the first four data

Group	Tg-DT status	No. of patients with Tg-DT calculated using	
		All available data	Only first four data
1	< 1 year	17 (4.0%)	20 (4.7%)
2	1–3 years	21 (4.9%)	22 (4.9%)
3	≧ 3 years	30 (7.0%)	26 (6.1%)
4	Negative value	69 (16.2%)	69 (16.2%)
5	Not calculated*	88 (20.7%)	88 (20.7%)
6	Tg not detectable	201 (47.2%)	201 (47.2%)

*Tg* thyroglobulin, *Tg-DT* thyroglobulin doubling time

*Patients with undetectable Tg levels and three or fewer detectable Tg levels. (Cited from Miyauchi et al. [9], with permission)

**Fig. 4:**
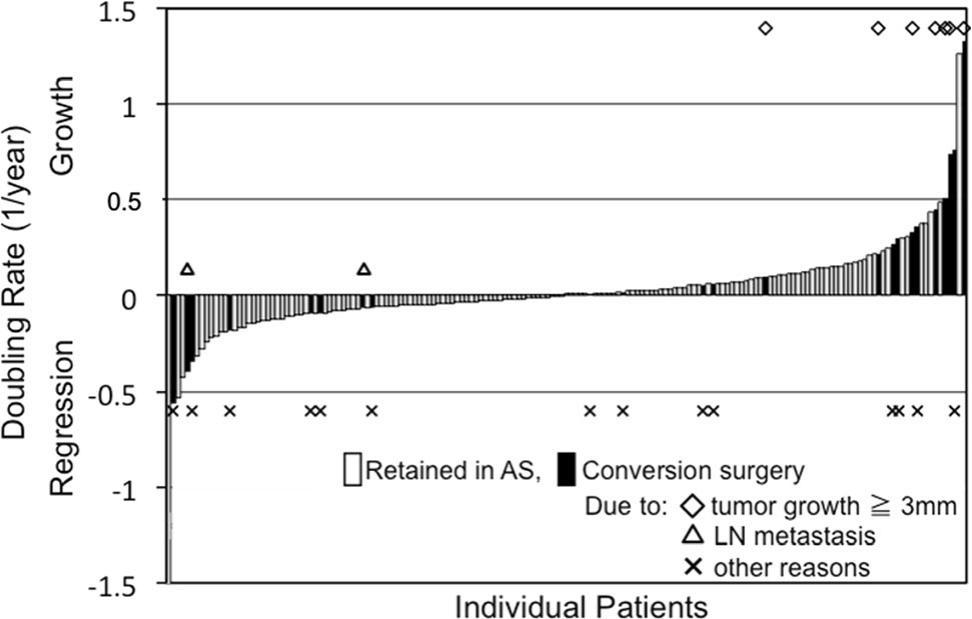
Tumor volume doubling rates in patients with papillary thyroid microcarcinoma during active surveillance. Positive value indicates tumor growth and large value indicates rapid growth. Negative value indicates decrease in tumor volume. (Cited from Miyauchi et al. [11], with permission)

**Fig. 5:**
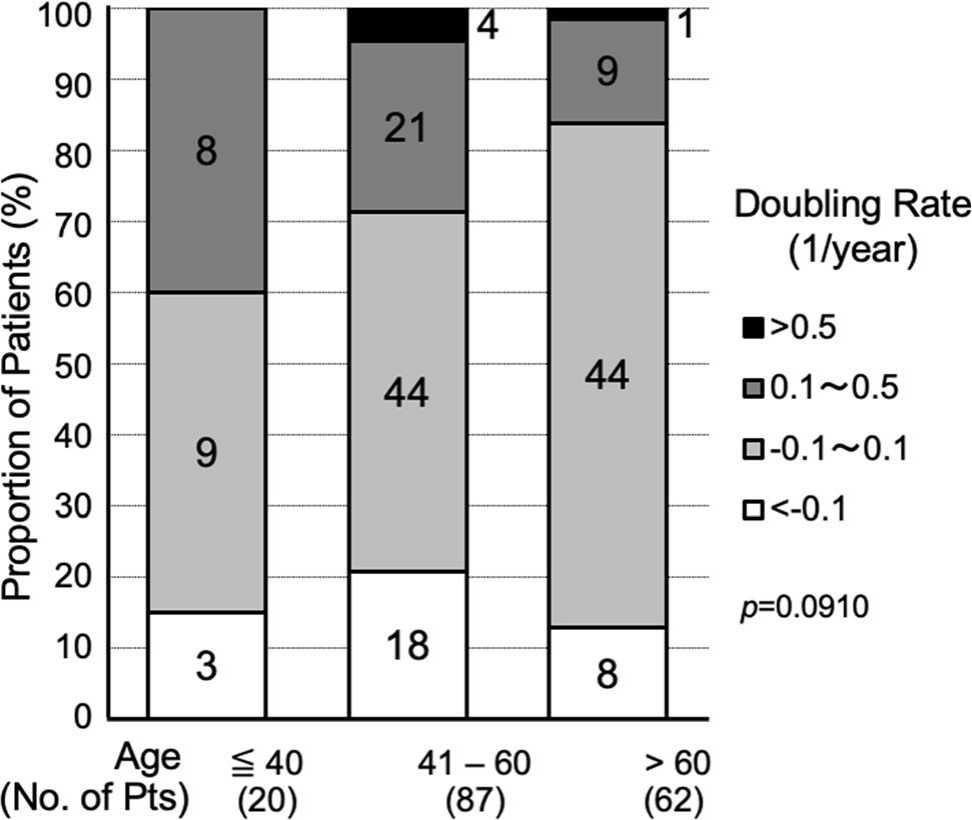
Distribution of tumor volume doubling rate in patients with papillary thyroid microcarcinoma during active surveillance according to age at presentation. (Cited from Miyauchi et al. [11], with permission)

DT is a well-validated way to analyze and express tumor volume kinetics over time. However, it has two major limitations. First, if some of the tumors lose their volume over time, their DTs are given in negative values. This causes the problem of discontinuity between positive values. Second, the magnitude of DT value is opposite to the magnitude of growth rate. Taking inverse of DT, these limitations are resolved [11]. I proposed calling this index doubling rate (DR), since it indicates number of doublings that occur in a unit time [11]. Using DR, we were able to show tumor volume kinetics in patients with papillary thyroid microcarcinoma (PTMC) on active surveillance (Fig. 4) [11]. During the active surveillance, only 3% of the patients showed moderate growth, 22% showed slow growth, 57% showed almost stable disease, and very interestingly 17% of the patients showed an evident decrease in their tumor volume over time. This was the first report on spontaneous regression of structural disease of thyroid cancer in a large patient cohort. The distribution DR in these patients of according to age at presentation, ≦ 40, 41–60 and > 60 years, revealed that the proportion of growing tumors decreased with age, and that the proportion of shrinking tumors was highest among middle-aged patients (Fig. 5) [11].

**Fig. 6:**
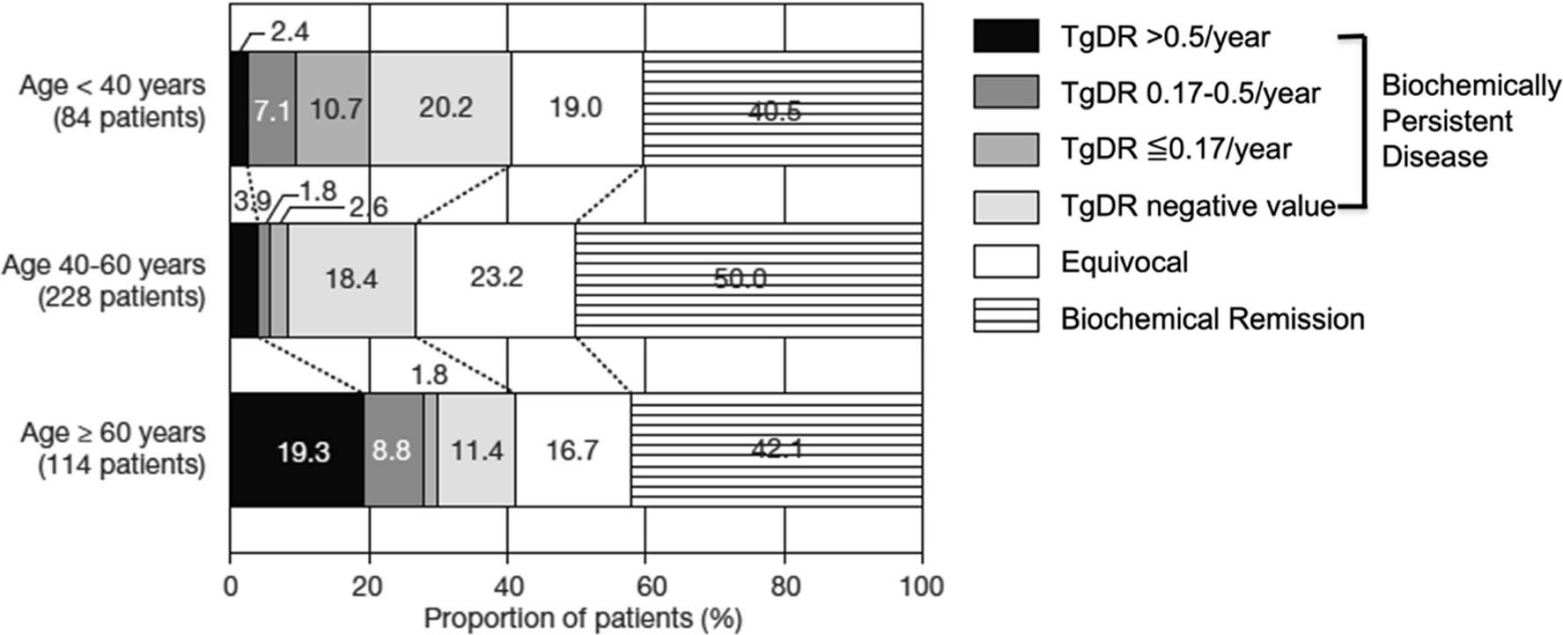
Postoperative thyroglobulin status and thyroglobulin doubling rate (Tg-DR) according to age in 426 patients with papillary thyroid carcinoma who underwent total thyroidectomy. Doubling time in the original was converted to doubling rate. (Cited from Miyauchi et al. [12], with permission)

The postoperative thyroglobulin status and thyroglobulin DR in 426 patients with PTC and negative thyroglobulin antibody who underwent total thyroidectomy across the similar age groups at surgery is shown in Fig. 6 [12]. The distribution pattern differed depending on the age group. The proportion of patients with moderate growth increased with age, and the proportion of slow growth was highest in young patients. Very interestingly, 18% of young patients and 20% of middle-aged patients showed negative thyroglobulin DR. In this cohort, 142 patients (33%) had BPD postoperatively, and proportion of patients with BPD was high in young and elderly patients and low in middle-aged (Table 2). As many as 72 (51%) of the patients with BPD showed negative thyroglobulin DR. This proportion was high in young and middle-aged patients (50% and 69%, respectively) and low in elderly patients (28%), suggesting regression in these patients.

**Table 2:** Biochemically persistent disease and thyroglobulin doubling rate according to age at surgery in 426 patients with papillary thyroid carcinoma who underwent total thyroidectomy

Age (years)	No. of Pts	Pts with BPD	%	Pts with negative TgDR value	Proportion among BPD (%)
< 40	84	34	40	17	50
40–60	228	61	27	42	69
≧ 60	114	47	41	13	28
Total	426	142	33	72	51

*BPD* biochemically persistent disease, *TgDR* thyroglobulin doubling rate. Doubling time in the original paper was converted to doubling rate. (Cited from Miyauchi et al. [12], with permission)

**Fig. 7:**
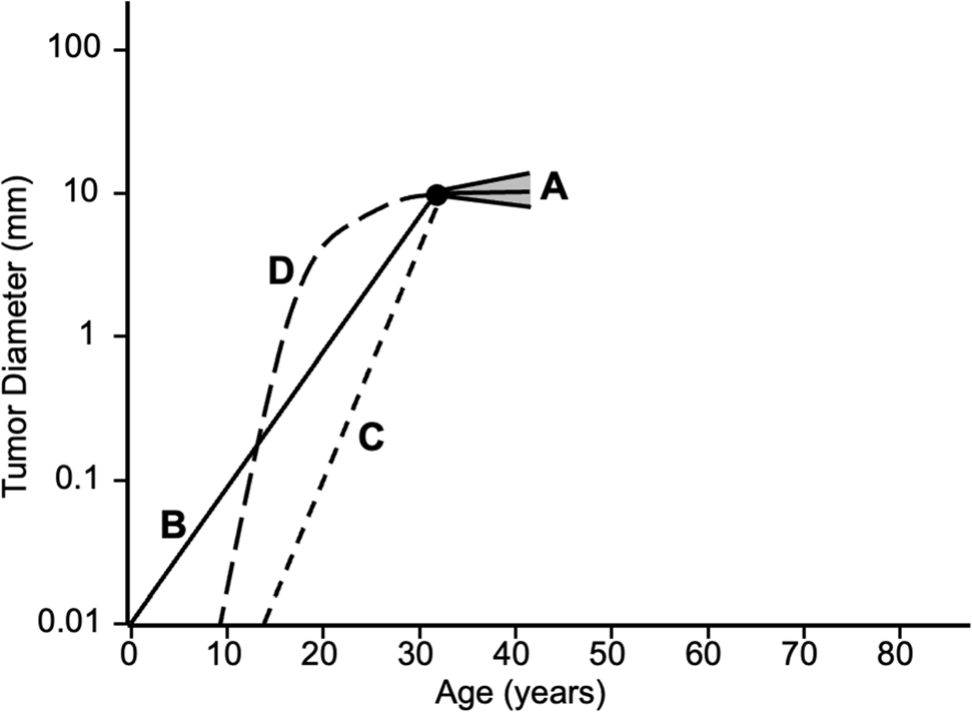
A schematic diagram of a 1 cm papillary thyroid microcarcinoma in a 30-year-old patient showing the observed range of tumor growth and regression. Line B: The hypothetical growth of cancer that occurs at birth. Line C: Growth of cancer that develops after birth. Line D: The most probable tumor growth pattern before presentation. (Cited from Miyauchi et al. [11], with permission)

We performed kinetic analyses on changes in serum tumor markers after surgery and on changes in tumor volume during active surveillance. Next, we wanted to know changes in tumor volume before presentation. We calculated hypothetical tumor volume-DR before presentation using the patient’s age and tumor size at presentation, presuming that a single 10 μm-diameter cancer cell was present at birth and grew at a constant rate [11]. You may argue that the origin of the cancer may be later than birth. Then, the growth should be faster than this estimate. The growth may not be even. If there are slow periods, there should be a much faster period for the cancer cell to reach the tumor size at presentation. Therefore, the hypothetical DR is the minimum growth rate required for a single cancer cell to reach the tumor size at presentation. The DR observed during active surveillance was significantly lower than the hypothetical DR before presentation (median 0.0/year vs. 0.5/year, *p* < 0.001) [11]. Figure 7 is a schematic diagram of a 1 cm PTMC in a 30-year-old patient showing the observed range of tumor growth and regression (Fig. 7). Line B shows the hypothetical growth of cancer that occurs at birth. If cancer develops after birth, the tumor should grow faster like line C. Tumor growth just before presentation should not differ significantly from the observed growth rate. Therefore, D is the most probable tumor growth pattern before presentation [11].

We studied 9 pediatric and adolescent patients with PTC who underwent total thyroidectomy and had high serum thyroglobulin after surgery but did not receive any dose of radioactive iodine because of their parents’ refusal. We found that the observed thyroglobulin DR was significantly lower than the hypothetical tumor volume DR before surgery (median 0.14/year vs. 2.0/year, *p* < 0.01). In addition, two of the patients had negative thyroglobulin DRs, suggesting spontaneous deceleration in growth and regression [13].

**Fig. 8:**
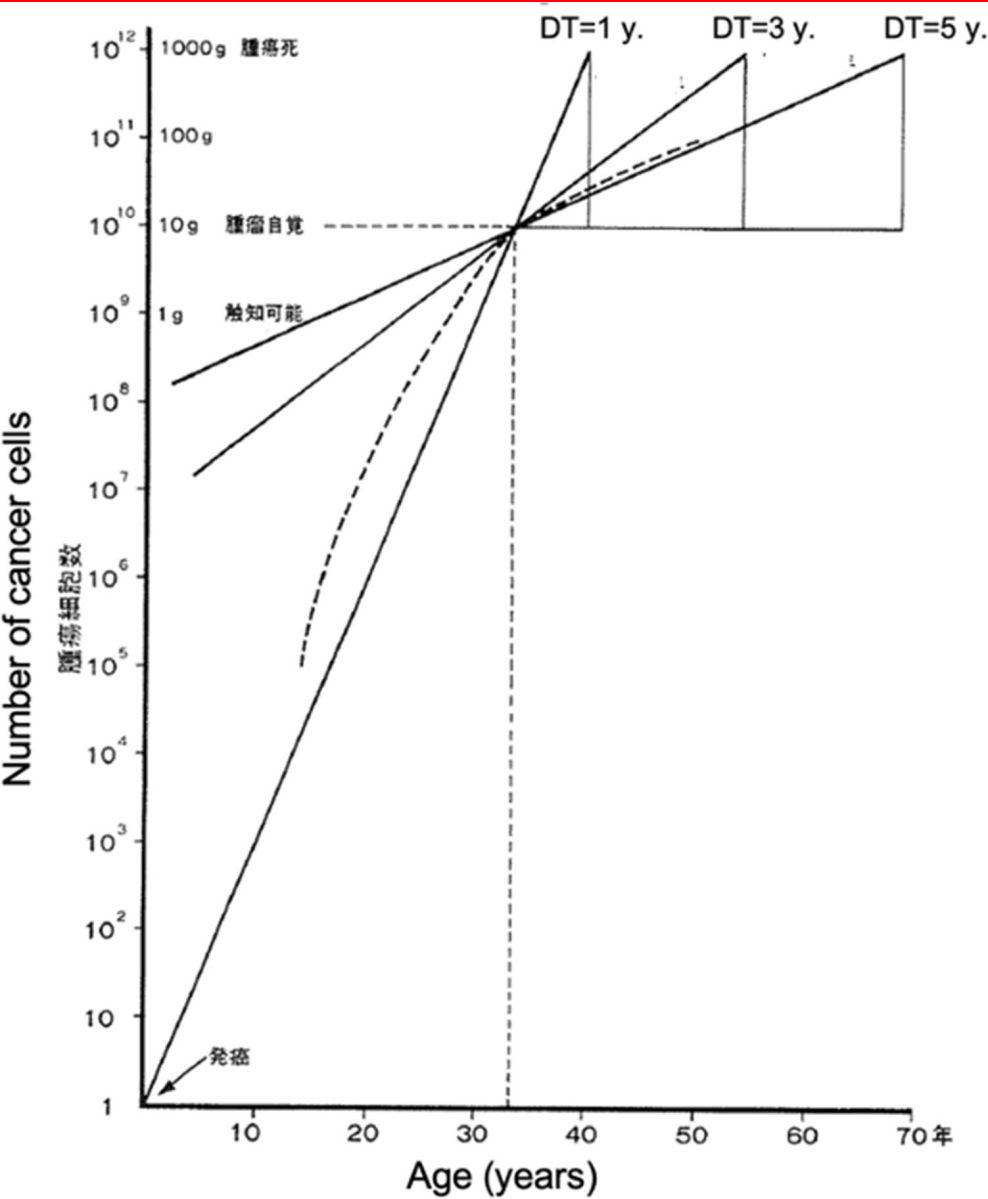
A schematic diagram of a 10 g medullary thyroid carcinoma in a 33-year-old patient. Calcitonin DT after surgery averaged 3–5 years. If you trace these growth lines back to birth, you will find big tumors at birth. That shouldn’t be the case. Therefore, the actual growth of the tumor should have been rapid at first and become slow later as shown in the broken line. (Cited from Miyauchi et al. [14], with permission)

A similar phenomenon was observed in MTC. Soon after our initial report on calcitonin DT, I recognized this and wrote a paper entitled “Chronology of Medullary Thyroid Carcinoma,” which was published on Journal of Japan Surgical Society [14]. Figure 8 is a schematic diagram of a 10 g MTC in a 33-year-old patient. Calcitonin DT after surgery averaged 3–5 years (Fig. 8). If you trace these growth lines back to birth, you will find big tumors at birth. That shouldn't be the case. Therefore, as shown by the broken line, the actual growth of the tumor should have been rapid at first and slow later. In addition, this also suggested that tumor growth would become even slower after surgery. Data supporting this assumption appeared later.

We studied 46 MTC patients who had BPD postoperatively [15]. We found that the observed calcitonin DR was significantly lower than the hypothetical tumor volume DR before surgery (median 0.17/year vs. 0.60/year, *p* < 0.001). We also found 9% of the patients having negative calcitonin DR, although these patients should have persistent disease. We studied other 26 MTC patients with BPD postoperatively who were followed for more than 10 years. In patients with hereditary disease, calcitonin DR in the later half period was significantly lower than that in the earlier half period [16]. Spontaneous decrease in growth rate in long term period was very common in patients with hereditary MTC. However, two patients showed sudden increase in DR [16].

As you know, spontaneous acceleration in growth rate is typically seen as anaplastic transformation, poor-differentiation or aggressive change of well-differentiated tumors. These are very easy to notice. On the contrary, spontaneous regression is not easy to notice. However, there are many fragments of evidences of this phenomenon. Microcalcification is a common ultrasound feature of PTMC. Pathology of PTMC often shows advanced hyalinization and calcification. I am sure that you have seen many patients with clinical PTC having calcification in the primary lesion and metastatic lymph nodes. These should be the evidences of regression at least locally.

## Summary

The evidences currently available are very limited. However, these data indicate the following: Growth slowdown and regression are very common phenomena in the natural history of PTMC, clinical PTC in young and middle-aged patients, and hereditary MTC. I think that this should be a reason why young PTC patients often suffered from advanced disease but had a good prognosis. DT and DR are very powerful tools to provide the most appropriate management for the patients with thyroid cancers. They are also useful tools to insight into the natural history of thyroid cancers. The biologic characteristics of cancers of the same name, such as PTC, are diverse and vary widely, especially with age. Knowing the natural history of thyroid cancer is essential for the best disease management of thyroid cancer.
